# Prediction of lung emphysema in COPD by spirometry and clinical symptoms: results from COSYCONET

**DOI:** 10.1186/s12931-021-01837-2

**Published:** 2021-09-09

**Authors:** Christina Kellerer, Rudolf A. Jörres, Antonius Schneider, Peter Alter, Hans-Ulrich Kauczor, Bertram Jobst, Jürgen Biederer, Robert Bals, Henrik Watz, Jürgen Behr, Diego Kauffmann-Guerrero, Johanna Lutter, Alexander Hapfelmeier, Helgo Magnussen, Franziska C. Trudzinski, Tobias Welte, Claus F. Vogelmeier, Kathrin Kahnert

**Affiliations:** 1 School of Medicine, Institute of General Practice and Health Services Research, Technische Universität München/Klinikum Rechts der Isar, Orleansstr. 47, 81667 Munich, Germany; 2 Institute and Outpatient Clinic for Occupational, Social and Environmental Medicine, Comprehensive Pneumology Center Munich (CPC-M), Ludwig-Maximilians-Universität München, Ziemssenstr. 1, 80336 Munich, Germany; 3 Department of Medicine, Pulmonary and Critical Care Medicine, University Medical Center Giessen and Marburg, Philipps-University Marburg, German Center for Lung Research (DZL), Baldingerstrasse, 35043 Marburg, Germany; 4 Department of Diagnostic and Interventional Radiology, University Hospital of Heidelberg, Heidelberg, Germany; 5 Translational Lung Research Centre Heidelberg (TLRC), Member of the German Center for Lung Research, Heidelberg, Germany; 6 Faculty of Medicine, University of Latvia, Raina bulvaris 19, Riga, 1586 Latvia; 7 Faculty of Medicine, Christian-Albrechts-Universität Zu Kiel, 24098 Kiel, Germany; 8 Department of Internal Medicine V – Pulmonology, Allergology, Respiratory Intensive Care Medicine, Saarland University Hospital, Kirrberger Straße 1, 66424 Homburg, Germany; 9 Airway Research Center North (ARCN), German Center for Lung Research (DZL), Pulmonary Research Institute at LungenClinic Grosshansdorf, Woehrendamm 80, 22927 Grosshansdorf, Germany; 10 Department of Internal Medicine V, University of Munich (LMU), Comprehensive Pneumology Center, German Center for Lung Research, Ziemssenstr. 1, 80336 Munich, Germany; 11 Comprehensive Pneumology Center Munich (CPC-M), German Center for Lung Research (DZL), Institute of Epidemiology, Helmholtz Zentrum München (GmbH) – German Research Center for Environmental Health, 85764 Neuherberg, Germany; 12 Translational Lung Research Centre Heidelberg (TLRC), Member of the German Center for Lung Research, Thoraxklinik-Heidelberg gGmbH, Röntgenstraße 1, 69126 Heidelberg, Germany; 13 Department of Pneumology, Hannover Medical School, Carl-Neuberg-Str. 1, 30625 Hannover, Germany

**Keywords:** Emphysema, CT scan, Decision trees, Random forest, Adaboost, COPD phenotypes

## Abstract

**Background:**

Lung emphysema is an important phenotype of chronic obstructive pulmonary disease (COPD), and CT scanning is strongly recommended to establish the diagnosis. This study aimed to identify criteria by which physicians with limited technical resources can improve the diagnosis of emphysema.

**Methods:**

We studied 436 COPD patients with prospective CT scans from the COSYCONET cohort. All items of the COPD Assessment Test (CAT) and the St George’s Respiratory Questionnaire (SGRQ), the modified Medical Research Council (mMRC) scale, as well as data from spirometry and CO diffusing capacity, were used to construct binary decision trees. The importance of parameters was checked by the Random Forest and AdaBoost machine learning algorithms.

**Results:**

When relying on questionnaires only, items CAT 1 & 7 and SGRQ 8 & 12 sub-item 3 were most important for the emphysema- versus airway-dominated phenotype, and among the spirometric measures FEV_1_/FVC. The combination of CAT item 1 (≤ 2) with mMRC (> 1) and FEV_1_/FVC, could raise the odds for emphysema by factor 7.7. About 50% of patients showed combinations of values that did not markedly alter the likelihood for the phenotypes, and these could be easily identified in the trees. Inclusion of CO diffusing capacity revealed the transfer coefficient as dominant measure. The results of machine learning were consistent with those of the single trees.

**Conclusions:**

Selected items (cough, sleep, breathlessness, chest condition, slow walking) from comprehensive COPD questionnaires in combination with FEV_1_/FVC could raise or lower the likelihood for lung emphysema in patients with COPD. The simple, parsimonious approach proposed by us might help if diagnostic resources regarding respiratory diseases are limited.

*Trial registration* ClinicalTrials.gov, Identifier: NCT01245933, registered 18 November 2010, https://clinicaltrials.gov/ct2/show/record/NCT01245933.

**Supplementary Information:**

The online version contains supplementary material available at 10.1186/s12931-021-01837-2.

## Background

Chronic obstructive pulmonary disease (COPD) is a common disorder with a high prevalence worldwide [1, 2]. Lung emphysema is an important phenotype of COPD, and the differentiation between emphysema- and airway-phenotypes is increasingly relevant for the management of the disease. Currently, computed tomography of the chest (CT) is the most precise method to detect, quantify and follow-up lung emphysema [3–5]. The differentiation between bronchitis and emphysema is important as emphysema shows functional characteristics different from those of chronic obstructive bronchitis [6–8], and patients with emphysema have partially different therapeutic options, such as lung volume reduction in case of severe hyperinflation [9–11], that are not relevant for predominant obstructive bronchitis. Current data show a protective effect of metformin on lung aging and thus on the development of emphysema, suggesting that in the future specific pharmacological therapeutic approaches might also become relevant in the treatment of emphysema [12]. Moreover, emphysema is associated with increased mortality risk and incidence of lung cancer [13, 14].

The clinical signs and symptoms of COPD can be quantified through questionnaires, such as the COPD Assessment Test (CAT) [1], modified Medical Research Council (mMRC) scale and St George’s Respiratory Questionnaire (SGRQ) [1]. For CAT it has been demonstrated that single items confer information on emphysema [8]. SGRQ as a whole is too time-consuming for application in specialist’s and non-specialist’s practices, but has not been studied for the value of single items. The selected items could be combined with spirometry, often available in clinical practice. It would be of interest to compare this setting with the potential gain from functional measurements available only to the specialist, such as CO diffusing capacity, a method that is informative regarding lung emphysema [7, 15].

We analysed a subset [8, 16] of the COSYCONET (COPD and Systemic Consequences-Comorbidities Network) COPD cohort [17], comprising patients with prospective CT scans evaluated for the presence of emphysema. The aim was to identify a minimal subset of criteria that would increase or decrease the likelihood for emphysema to support clinical decision making for further diagnostic testing, such as CT. For this purpose, we used tree-based algorithms, either as single trees, or as statistical ensembles of trees. Via these approaches, we evaluated different sets of diagnostic criteria, ranging from single clinical symptoms to functional data available only to the pulmonary specialist.

## Methods

### Study cohort

Using the clinical and functional assessments in the multi-center COPD cohort COSYCONET [17], the present analysis was based on a subproject involving CT scans in inspiration and expiration under standardized conditions (for detailed information see Additional file 1). CT scans were performed around the time point of the third follow-up visit (visit 4), thus the functional and clinical data of this visit were used for analysis. At visit 4, 1427 of initially 2741 patients with COPD recruited at visit 1 still participated in COSYCONET, among these 1176 of spirometric GOLD grades 1–4. Of these 1427 patients, 518 participated in the CT substudy and had CT scans that could be evaluated qualitatively for the presence of either an airway-dominated or an emphysema-dominated phenotype. Among these patients, 436 showed GOLD grades 1 to 4 [1] at visit 4 and represented the present study population. CTs were assessed in 16 study centers, and their analysis was performed by experienced radiologists in the COSYCONET centre for image evaluation (University of Heidelberg); details can be found in the Additional file 1. The binary emphysema score served as primary indicator of the COPD lung phenotype in all evaluations.

### Assessments

Clinical history was assessed via standardized questionnaires [17], and clinical signs and symptoms via the instruments CAT [1], mMRC [1] and SGRQ [18]. Diagnosis of comorbidities were taken from the patients’ reports of physician-based diagnoses. The lung function assessments evaluated comprised spirometry and diffusing capacity for carbon monoxide (CO), which were performed according to SOPs following international guidelines and recommendations [17, 19]. The parameters used in the present study were the forced expiratory volume in one second (FEV_1_), forced expiratory volume (FVC), their ratio FEV_1_/FVC, the transfer factor for CO (TLCO) and the transfer coefficient for CO (KCO). Predicted values were taken from the Global Lung Function Initiative (GLI) [20, 21].

### Statistical analysis

Mean values and standard deviations (SD) were used to describe the distribution of quantitative data. Qualitative data is presented as absolute and relative frequencies. Hypothesis testing of group differences was performed by t-tests and Chi-squared tests, as appropriate. In order to obtain results that were best suited for potential application in clinical practice, we concentrated on single decision trees constructed with the exhaustive CHAID algorithm as implemented in SPSS [22]. Only binary branching was allowed to keep the trees simple, and all results given were based on tenfold cross validation and Bonferroni corrections to minimize errors. The analyses used all items of the questionnaires and optionally either spirometric, or spirometric and CO diffusing capacity data, with the aim to offer a wide panel of diverse information which was simple enough to be obtained in clinical practice. Thus, we selected the parameters offered to the algorithm under clinical and practical perspectives, but the final selection was based on statistical significance.

The trees’ predictive performance and the relevance of the selected predictors were explored by comparison with the respective results of two commonly used and well-performing reference methods. The first was the Random Forest approach, which is based on the construction of a random ensemble of decision trees [22, 23]. We used the standard settings of 500 trees and the number of variables chosen for splitting per node to be based on the square root of the number of variables. For this purpose, the package “randomForest” of the statistical software R (Version 4.0.2) was used [23, 24]. The second approach was the AdaBoost procedure that aims at the construction of a strong predictor by successive refinement of a set of weak predictors [24–26]. This method was realized using the packages “adabag” and “caret” from R [26]. To compare the methods we show the variables selected by these procedures in the rank order of importance according to the criterion of the mean decrease in accuracy (Random Forest) and the importance measure defined in the AdaBoost procedure, whereby the overall classification error refers to tenfold cross-validation in the case of AdaBoost and CHAID. All other computations were performed by SPSS Version 26 (IBM Corp., Armonk, NY, USA). Exploratory hypothesis testing was performed at two-sided significance levels of 0.05.

## Results

### Study cohort

Of the 1427 patients participating in visit 4, 436 patients were eligible for analysis by having data on the presence of emphysema from the CT scoring and being categorized as COPD grades 1 to 4 (Table 1). The statistical comparison with patients not included in the CT analysis (Table 1) showed significant differences regarding FEV_1_, FVC, in terms of %predicted, moreover the distribution over GOLD grades and groups. All differences, however, were small, indicating that patients with CT showed slightly less severe COPD than patients without CT. Of the patients with CT, 185 showed an emphysema-dominated, and 251 an airway-dominated phenotype of COPD.

**Table 1:** Patient characteristics

Variables	Group without CT	Group with CT	p-value
Sex (m/f)	448/292	271/165	0.583
Age (years)	68.0 ± 8.0	66.2 ± 8.2	p < 0.001
BMI (kg/m^2^)	26.7 ± 5.2	26.7 ± 4.9	0.898
Packyears	46.7 ± 37.6	46.8 ± 36.8	0.950
Smok. never, ex-, act	51/567/121	27/319/90	0.178
FEV_1_ %predicted	51.1 ± 18.6	55.4 ± 18.7	p < 0.001
FVC %predicted	76.1 ± 18.9	82.6 ± 20.3	p < 0.001
FEV_1_/FVC	0.51 ± 0.11	0.51 ± 0.11	0.475
TLCO %predicted	56.1 ± 22.6	59.6 ± 22.7	0.015
KCO %predicted	64.3 ± 25.6	65.2 ± 21.8	0.531
GOLD grades 1/2/3/4	54/310/283/93	49/211/137/39	0.003
GOLD groups ABCD	237/163/113/222	170/86/80/99	0.014

Comparison of the subgroup with CT scans (study population) and the patients without CT scans from COSYCONET that were participants at the time of the CT scans. Statistical comparisons were performed with either t-tests or Chi-square contingency tables, as appropriate

### Tree-based prediction of emphysema

In the decision and classification trees we focused on those combinations of values that showed either the greatest likelihood for emphysema dominance or the greatest likelihood for airway dominance (i.e. against emphysema) according to CT. Thus, we put the emphasis on the combinations of values yielding the best predictions, neglecting all other combinations that showed only slight changes in the likelihood of emphysema. Decision trees allow to identify not only values that are informative but also values that are not informative [22]. This was done for the set of all questionnaire items (CAT, SGRQ, mMRC), moreover their combination with spirometric parameters including FEV_1_/FVC.

**Fig. 1:**
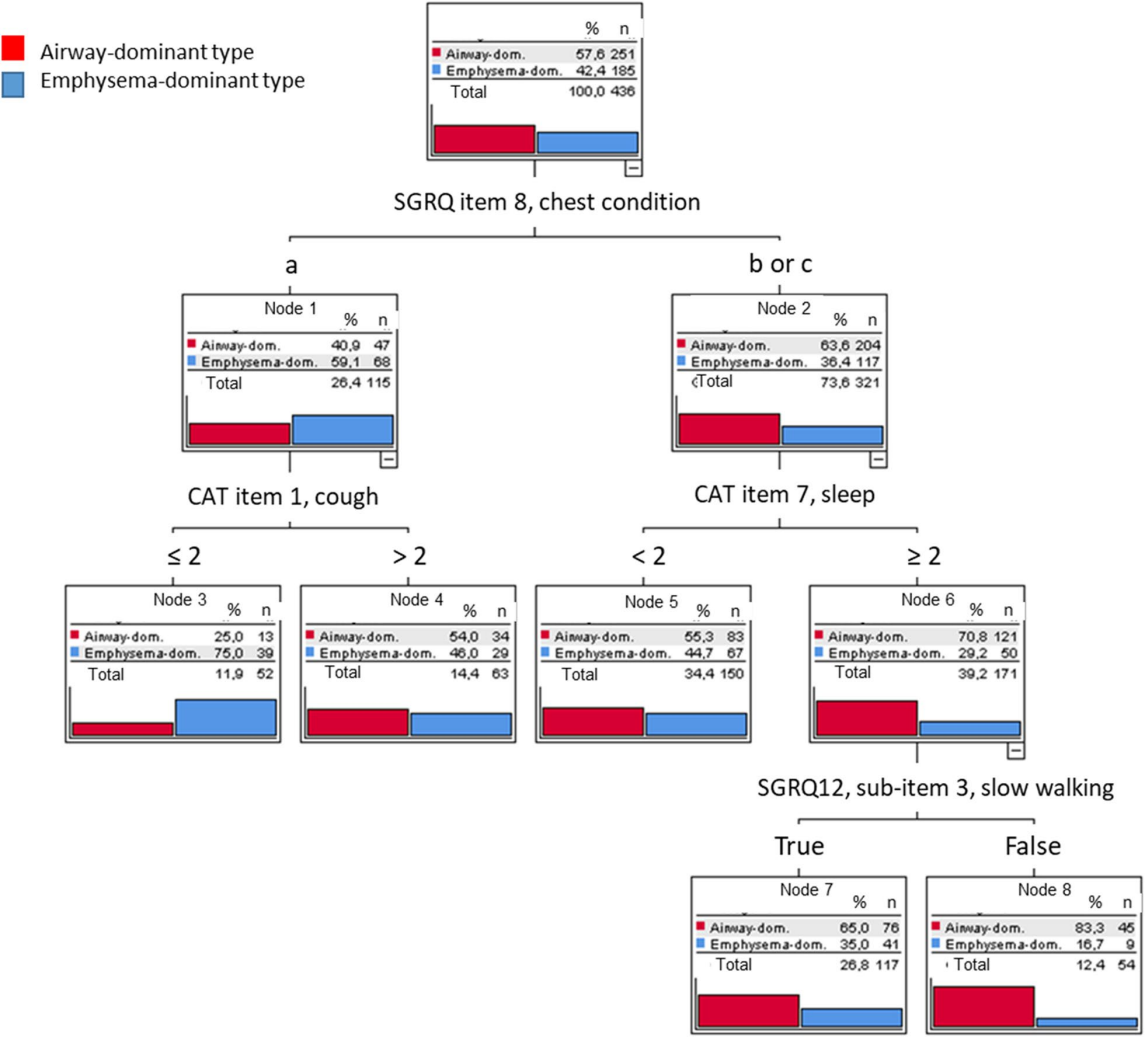
Decision tree derived from the inclusion of CAT, SGRQ and mMRC. Only CAT items 1 and 7 as well as SGRQ items 8 and SGRQ 12 sub-item 3 were selected as significant predictors. Item 8 of the SGRQ is the question „How would you describe your chest condition?” with following answer options a) Causes me a lot of problems or is the most important problem I have, b) Causes me a few problems and c) Causes no problem. Please note the large differences in the distribution of diagnoses, whereby the final nodes 4, 5 and 7 were scarcely informative compared to the prior values (comprising 327 patients). In contrast, nodes 3 and 8 were informative (comprising 106 patients). The odds ratio for emphysema corresponding to node 3 was 4.06

**Fig. 2:**
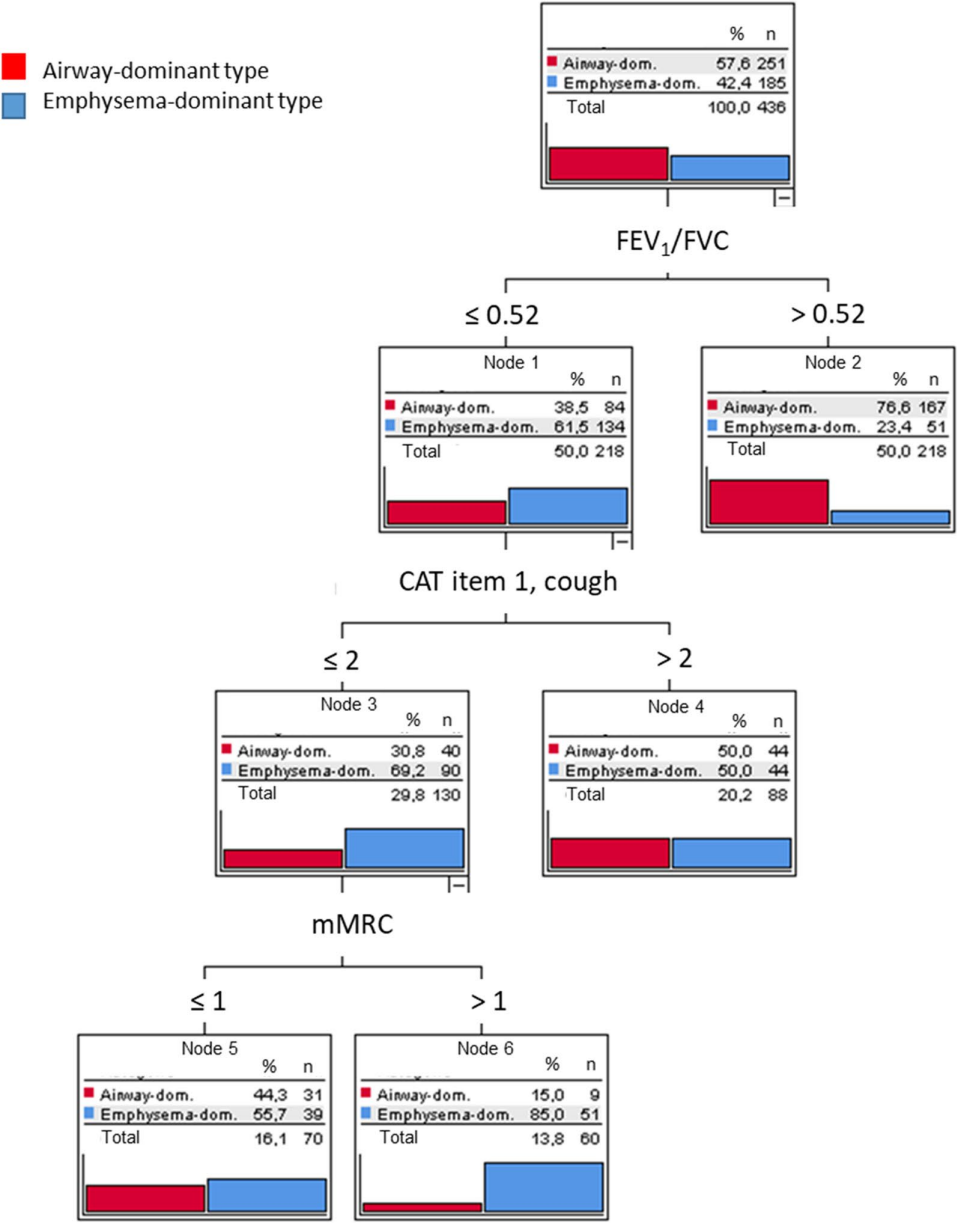
Decision tree derived from the inclusion of CAT, SGRQ, mMRC and FEV_1_/FVC. Only item 1 from CAT, mMRC and FEV_1_/FVC were selected as significant predictors. Please note the large differences in the distribution of diagnoses; nodes 2 and 6 were maximally informative (n = 278 patients), nodes 4 and 5 less informative (n = 158 patients). The odds ratio for emphysema corresponding to node 6 was 7.69

The respective trees are shown in Figs. 1 and 2. It can be seen that odds ratios for emphysema ranged above 4 and that the combination of CAT item 1 (cough), mMRC and FEV_1_/FVC yielded an odds ratio of more than 7, while odds ratios for the absence of emphysema tended to be lower. Importantly, the trees demonstrate that some combinations of individual values, i.e. combinations of binary partitions and respective definitions of patient subgroups, were highly informative compared to baseline but other combinations not, irrespective of the fact that the partitions at each node were statistically significant. For practical purposes, Fig. 4 summarizes the results of the decision trees in a single diagram, whereby we selected the nodes showing the maximum odds ratios for the emphysema- or airway-dominant phenotype.

**Fig. 3:**
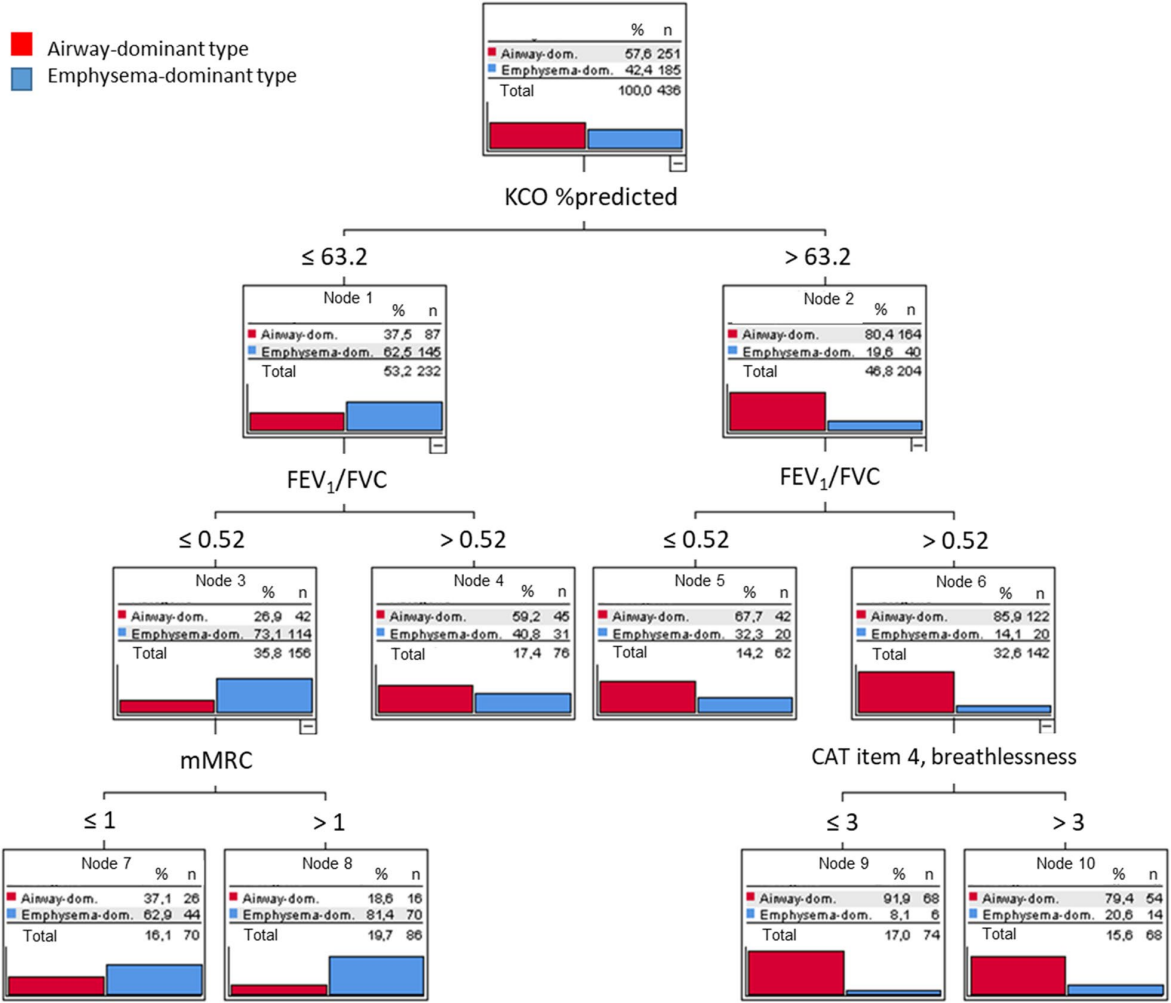
Decision tree derived from the inclusion of CAT, SGRQ, mMRC, spirometric, and CO diffusing capacity parameters. Only item 4 from CAT, mMRC, KCO %predicted and FEV_1_/FVC were selected as significant predictors. Please note the large differences in the distribution of diagnoses; nodes 8 and 9 were maximally informative (n = 160 patients), nodes 4, 5, 7 and 10 less informative (n = 276 patients). The odds ratio for emphysema corresponding to node 8 was 5.51

**Fig. 4:**
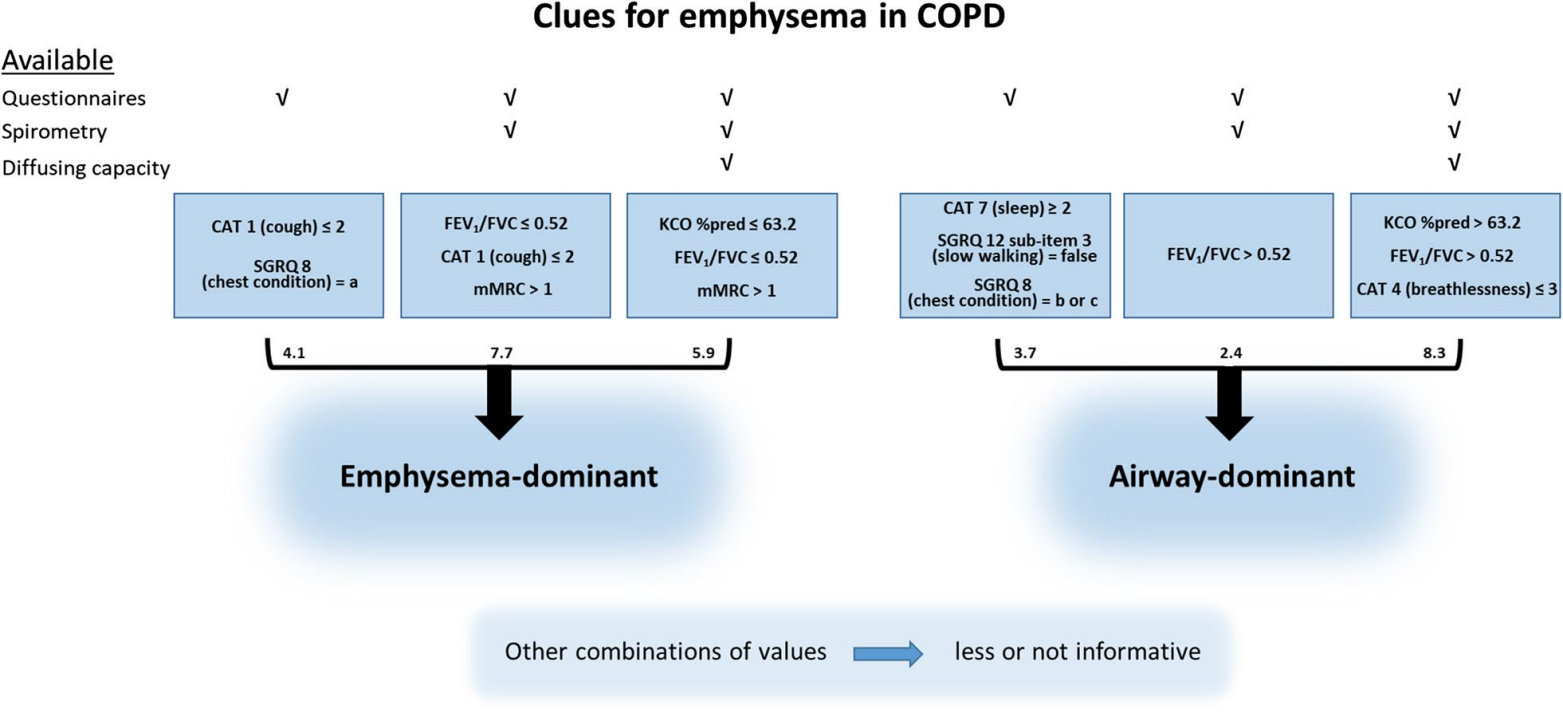
Summary of the results of the decision trees obtained for questionnaire data in combination with spirometry and CO diffusing capacity in terms of the transfer coefficient KCO. Only those conditions are shown that maximize the odds ratios for each of the two phenotypes. The conditions favouring either the emphysema- or the airway-dominated phenotype are given within the boxes, while the numbers indicate the respective odds ratios that can be derived from Figs. 1, 2 and 3. The interpretation of the questionnaire items is identical with that of the original CAT, SGRQ or mMRC questionnaires, the %predicted values for KCO refer to GLI [21]

To assess the prediction of emphysema including CO diffusing capacity, we repeated the sequence of analyses with the data from clinical history/questionnaires and spirometry but additionally diffusing capacity. The respective tree is shown in Fig. 3 and the results are included in Fig. 4. KCO %predicted was the primary decision parameter, followed by FEV_1_/FVC, while CAT item 4 (breathlessness) and mMRC were additionally relevant. It should be noted that high values of KCO and FEV_1_/FVC without the CAT item 4 already resulted in a high odds ratio for the airway-dominant type, as indicated in node 6 in Fig. 3.

### Comparison of decision trees with machine learning results

The machine learning methods Random Forest and AdaBoost are often used to improve the predictive performance of classification trees. We used these procedures to assess whether the overall accuracy was similar to that of the single trees. If it would be markedly higher, this would point to a classification problem of higher complexity that cannot be adequately solved by a classification tree. The results are shown in the Additional file 1: Table S1, indicating that the sets of important variables contained those selected in the single decision trees. Moreover, the overall errors were similar in both the machine learning approaches and the single decision trees. It should be noted that the overall error comprises the combinations of values that were not informative as illustrated in Figs. 1, 2, 3, thereby overestimating the error obtained when restricting to the cases with high odds ratios shown in Fig. 4.

## Discussion

The present analysis had the aim to identify criteria by which physicians with limited technical resources regarding respiratory diseases can improve the diagnosis of emphysema in COPD patients. This could be achieved by a small set of questions combined with spirometry, whereby the responses and values indicated either a markedly elevated or a lowered likelihood for the emphysema-dominated phenotype. This might strengthen the rationale for performing quantitative assessment of the lung with CT. We evaluated all single items of the CAT and SGRQ, as well as the mMRC, supplemented by spirometric data, and for comparison also CO diffusing capacity. In the decision trees, three items of the CAT, two items of the SGRQ, as well as the mMRC were informative. Among spirometric parameters, FEV_1_/FVC was most informative, and among the parameters of CO diffusing capacity, the transfer coefficient (KCO) expressed as %predicted. This was evident in the decision trees from those combinations of values that maximally raised the likelihood for an emphysema- or airway-dominated phenotype. In combination with FEV_1_/FVC, odds ratios ranged up to values of more than 7. Using diffusing capacity, the odds ratio for the airway-dominated phenotype reached a similar value. Our results demonstrate that a few anamnestic questions plus spirometric data can provide significant evidence on the presence of lung emphysema which subsequently can be substantiated by ordering a CT scan.

The most precise diagnosis and quantification of lung emphysema is achieved by chest CT but due to limited availability and cost restrictions, this is not yet clinical routine. As the identification of patients with emphysema is relevant for therapeutic decisions and interventions, all information supporting a well-founded referral for quantitative CT is helpful. If selected questionnaire items are associated with emphysema, as demonstrated for the CAT [8], this is particularly useful, since long questionnaires are difficult to implement in the busy routine of a family physician’s practice. With regard to CAT and mMRC, the present results were consistent with the previous observations of correlation patterns [8] while for the SGRQ single item data are not available. The single item approach was essential in finding concise algorithms that might be particularly suited for a family physician’s daily routine with limited diagnostic resources regarding respiratory diseases (see Fig. 4).

We focused on patients with either a high or a low likelihood of emphysema. Only in these cases, we expected that algorithms based on few simple questions can be efficient. Conversely, the decision trees indicated, that in about half or more of the cases the changes in likelihood were low. Such patients, in whom an informed decision well supported with the available data, would need further diagnostic evaluation, i.e. referral to a specialist, and we consider their identification an advantage. When computing overall sensitivity for emphysema by averaging over all conditions, values were 21.1%, 72.4%, and 61.6% for questionnaire items alone, questionnaires combined with spirometry, and the combination of questionnaires, spirometry and KCO, respectively. Conversely, the sensitivity for the airway-dominated type was 94.8%, 66.5%, and 83.3%, respectively.

To estimate the maximum accuracy, we evaluated the gain by adding data of CO diffusion capacity as typically obtained in a pulmonologist’s practice. As expected, CO diffusing capacity conferred the primary information on the presence of emphysema, in line with previous results delineating the cumulative value of spirometry, diffusing capacity and bodyplethysmography [7]. From Fig. 4 and the comparison of the decision trees shown in Figs. 2 and 3, it appeared that the major benefit from KCO referred to the recognition of the airway-dominated phenotype.

Decision trees are susceptible to overfitting which we tried to reduce by using tenfold cross validation. In order to check the results, we additionally employed machine learning methods that rely on ensembles of trees or a sequence of consecutively refined trees. For this purpose, we performed a sensitivity analysis, using the Random Forest and the AdaBoost approach. This related but different approach confirmed that the questionnaire items and functional parameters revealed as important comprised the variables identified in the single decision trees. We did not use this as primary analysis as it does not result in directly comprehensible trees.

Figure 4 summarizes the clues for an emphysema- versus airway-dominated phenotype in a comprehensive and easily applicable form. For example, a low symptom burden from cough (CAT item 1 ≤ 2) combined with shortness of breath at common exercise levels (mMRC > 1) and an impaired ratio FEV_1_/FVC (≤ 0.52) raised the likelihood of emphysema by a factor of about 7.7. This underlines that easily obtained information can be sufficient for an informed decision, for example regarding the order of a CT-scan, independent from other diagnostic intentions, for example regarding lung cancer. It might be surprising that smoking history did not play a role in the decision trees, but this was probably due the fact that we included only patients with an established diagnosis of COPD. Moreover, when FEV_1_/FVC was included, the values of FEV_1_%predicted and correspondingly GOLD grades did not provide additional significant information, at least with the maximum tree depths of 3 which we fixed in order to keep the results robust and interpretable. For clinical practice, the COPD phenotype is of interest. For example, patients with advanced emphysema may benefit from lung volume reduction procedures in case of severe hyperinflation [11], and recent data described a relationship between cigarette smoke-induced oxidative stress and inflammation, leading to enhanced lung aging, apoptosis and emphysema [12]. The data also provided evidence for protective effects of metformin on the progression of emphysema [12]. These findings point towards potential future treatment options for emphysema and emphasize the need to determine the dominant phenotype.

## Limitations

The analysis was based on a subset of the COSYCONET cohort, and there were slight differences between patients having a CT scan versus those having no scan. In principle, differences between this cohort and typical primary care populations are possible but there are no sufficient data on this. At least, the percentage of emphysema-dominated type (42.4%) was similar to those observed in other large COPD studies [27, 28]. There is no evidence that possible differences between populations affected the validity of the decision trees, especially the identification of informative vs non-informative nodes. Therefore, further studies with typical populations of clinical practices and different a priori likelihood for COPD and emphysema would be useful. It should be kept in mind that our analysis refers to patients who have already the diagnosis of COPD, therefore symptoms such as slow walking and sleep disturbance are to be interpreted on this background. Without prior diagnosis of COPD, these symptoms will be less indicative of emphysema; our study aimed at providing simple and easy diagnostic help for specific conditions not in general. The limited size of the dataset also limited the maximum depth of the decision trees, as we followed the standard requirement of a minimum number of 50 patients in each node. This was no disadvantage in terms of usability, as trees with many levels would be less applicable than simple trees. In addition, we consider the risk of overfitting as minor, as we performed cross-validation and the variables identified in the single decision trees were among the variables identified in the machine learning algorithms. Another limitation is that our findings were based on a secondary analysis, thus the findings need to be validated in a confirmatory study, ideally in a family physicians’ setting with limited diagnostic resources regarding respiratory diseases. Due to the selection of patients participating in a clinical study such as COSYCONET, it cannot be anticipated, how robust the suggested approach will work in clinical settings involving a larger spectrum of differential diagnoses that have an impact on symptoms, such as cardiac diseases. It therefore might be a next step to perform a similar analysis in family physicians’ cohorts.

## Conclusions

Emphysema is an important phenotype of COPD and commonly diagnosed via chest CT scans involving additional costs and radiation exposure. Provided that a diagnosis of COPD has been established, the use of few single items of COPD questionnaires in combination with FEV_1_/FVC significantly raised or lowered the likelihood of an emphysema- versus airway-dominated COPD phenotype in a large proportion of patients. The simple, easy to apply criteria proposed by us might be useful in clinical practice for the decision of ordering a CT scan, particularly in non-pulmonary specialist settings, such as family physicians. The second result was that patients with answers and FEV_1_/FVC values that did not markedly change the likelihood could also be identified, which might be helpful in the decision to refer them to pulmonary specialists.

## Supplementary Information

**Additional file 1: Table S1. **Results of the Random Forest and AdaBoost procedures for different sets of variables included. The table shows the variables in the rank order of importance according to the criterion of the mean decrease in accuracy (Random Forest) and the importance measure defined in the AdaBoost procedure. The overall classification error refers to 10-fold cross-validation in the case of AdaBoost and CHAID. SGRQ4 “I have attacks of wheezing”, SGRQ5 “How many attacks of chest trouble did you have during the last year?”, SGRQ8, “How would you describe your chest condition?”, SGRQ11 sub-item 5 “I have become frail or an invalid because of my chest”, SGRQ12 sub-item 1 “I take a long time to get washed or dressed”, SGRQ12 sub-item 3 “I walk slower than other people, or I stop for rests”, SGRQ12 sub-item 4 “Jobs such as housework take a long time, or I have to stop for rests”, SGRQ13 sub-item 1 “I cannot play sports or games”, SGRQ14 “How does your chest trouble affect you? **Table S2. **Technical details of the CT assessment. The acquisition protocol is given in the upper part and details on the scanner models in the lower part of the table. *Vendor-specific generic names for Siemens/GE/Philips. **Table S3. **Items from the “St. George’s Respiratory Questionnaire” (SGRQ) that turned out to be informative in the single decision trees.** Table S4. **Items from the COPD Assessment test (CAT).** Table S5. **Modified Medical Research Council (mMRC) scale. This self-rating questionnaire is used to measure the degree of disability that breathlessness poses on day-to-day activities on a scale from 0 to 4.

## Data Availability

The basic data are part of the German COPD cohort COSYCONET (www.asconet.net/) and available upon request. There is a detailed procedure for this on the website of this network. Specifically, the data can be obtained by submission of a proposal that is evaluated by the steering committee. All results to which the manuscript refers, are documented appropriately in the text, figures or tables.

## Abbreviations

CAT: COPD Assessment Test
COPD: Chronic obstructive pulmonary disease
CT: Computed tomography
FEV1: Forced expiratory volume in one second
FVC: Forced expiratory volume
mMRC: Modified Medical Research Council scale
SD: Standard deviation
SGRQ: St George’s Respiratory Questionnaire
