# Supplementary material for: Prediction of lung emphysema in COPD by spirometry and clinical symptoms: results from COSYCONET
Source: Respir Res. 2021 Sep 9;22:242. doi: 10.1186/s12931-021-01837-2 (PMC8427948; doi:10.1186/s12931-021-01837-2)
Supplement: Supplementary file 1 — Additional file 1: Table S1. Results of the Random Forest and AdaBoost procedures for different sets of variables included. The table shows the variables in the rank order of importance according to the criterion of the mean decrease in accuracy (Random Forest) and the importance measure defined in the AdaBoost procedure. The overall classification error refers to 10-fold cross-validation in the case of AdaBoost and CHAID. SGRQ4 “I have attacks of wheezing”, SGRQ5 “How many attacks of chest trouble did you have during the last year?”, SGRQ8, “How would you describe your chest condition?”, SGRQ11 sub-item 5 “I have become frail or an invalid because of my chest”, SGRQ12 sub-item 1 “I take a long time to get washed or dressed”, SGRQ12 sub-item 3 “I walk slower than other people, or I stop for rests”, SGRQ12 sub-item 4 “Jobs such as housework take a long time, or I have to stop for rests”, SGRQ13 sub-item 1 “I cannot play sports or games”, SGRQ14 “How does your chest trouble affect you? Table S2. Technical details of the CT assessment. The acquisition protocol is given in the upper part and details on the scanner models in the lower part of the table. *Vendor-specific generic names for Siemens/GE/Philips. Table S3. Items from the “St. George’s Respiratory Questionnaire” (SGRQ) that turned out to be informative in the single decision trees. Table S4. Items from the COPD Assessment test (CAT). Table S5. Modified Medical Research Council (mMRC) scale. This self-rating questionnaire is used to measure the degree of disability that breathlessness poses on day-to-day activities on a scale from 0 to 4. [file 12931_2021_1837_MOESM1_ESM.docx]

**Additional file 1**

**Radiological evaluation**

The low-dose CT protocol included an inspiratory and expiratory scan of the entire lung with identical examination protocols performed on contemporary, commercially available clinical CT scanners (at least 40-row detector arrays). The standardized non-enhanced, low-dose chest CT protocol was derived from clinical standards and comprised end-inspiratory and end-expiratory acquisitions of the entire lung. The definition of the protocol was based on the recommendations of the German Radiological Society (DRG) using a thin slice collimation of 0.6 mm, a pitch of 0.6-1.0, a tube potential of 120 kVp and a tube current of 35 effective mAs for most scanner types [1]. As the study was performed on different scanner types, different generic CT acquisition protocols were required (see Table S2). The maximum effective dose of inspiratory and expiratory CT scans together was kept below 3.5 mSv.

An integrative part of the study was the goal of achieving a maximum of quality assurance. Therefore, the CT scanners were not only subject to standard procedures including daily calibration for air and every three months calibration for water during routine maintenance, but were also assessed regularly to phantom scans (Catphan®, The Phantom Laboratory, Salem, NY, USA). Table S2 shows the details on CT image reconstructions including three-dimensional datasets with thin slice thickness and smoothing as well as edge-enhancing algorithms. The central coordinating centre in Heidelberg (COSYCONET image repository) performed the scientific evaluation of all image data used for analysis. Images were evaluated visually using a multimodal OsiriX-based (OsiriX 64-bit, Pixmeo SARL, Geneva, Switzerland) post-processing workstation comprising an iMac 27” (Apple Inc., Cupertino, California, USA) and two 21” certified medical image displays (Eizo Nanao Corporation, Hakusan, Ishikawa, Japan). All examinations were read separately by experienced thoracic radiologists blinded to the clinical data. The semi-quantitative, visual analysis of CT data was performed on the basis of the modified guidelines of the COPD Gene CT Workshop Group [2].

| **Sets of**  **variables included** | **Random Forest** | **Adaboost** | **Overall classification error** | | |
| --- | --- | --- | --- | --- | --- |
|  |  |  | **Random Forest** | **AdaBoost** | **Single CHAID** |
| Questionnaires CAT SGRQ mMRC | mMRC, SGRQ12 sub-item 3, SGRQ8, CAT 7, SQRQ12 sub-item 1, SGRQ14, SGRQ11 sub-item 5, SGRQ12 sub-item 4, CAT 1, 4, 5 | CAT 7, 3, 2, 1, 8, 4, 5, SGRQ4, SGRQ8, SGRQ14, mMRC, SGRQ5, SGRQ13 sub-item 1 | 36.0 % | 44.7 % | 36.5 % |
| Questionnaires CAT SGRQ mMRC, Spirometry | FEV_1_/FVC, FEV_1_, mMRC, CAT 7, SGRQ 8, SGRQ11 sub-item 5, FVC, SGRQ14, SGRQ12 sub-item 3 | FEV_1_/FVC, FEV_1_, FVC, CAT 7, SQRQ14, CAT 2, 1, mMRC, CAT 4, 5 | 29.8 % | 36.7 % | 31.0 % |
| Questionnaires CAT SGRQ mMRC, Spirometry,  CO diffusing capacity | KCO, FEV_1_/FVC, TLCO, FEV_1_, mMRC, SGRQ8, CAT 7, SGRQ14, CAT 5, SGRQ12 sub-item 3 | FEV_1_/FVC, FVC, KCO, TLCO, FEV_1_, CAT 7, 3, SGRQ5, CAT 8, 5, 1, 4, 6, 2 | 29.1 % | 36.0 % | 25.9 % |

**Table S1. Results of the Random Forest and AdaBoost procedures for different sets of variables included.** The table shows the variables in the rank order of importance according to the criterion of the mean decrease in accuracy (Random Forest) and the importance measure defined in the AdaBoost procedure. The overall classification error refers to 10-fold cross-validation in the case of AdaBoost and CHAID. SGRQ4 “I have attacks of wheezing”, SGRQ5 “How many attacks of chest trouble did you have during the last year?”, SGRQ8, “How would you describe your chest condition?”, SGRQ11 sub-item 5 “I have become frail or an invalid because of my chest”, SGRQ12 sub-item 1 “I take a long time to get washed or dressed”, SGRQ12 sub-item 3 “I walk slower than other people, or I stop for rests”, SGRQ12 sub-item 4 “Jobs such as housework take a long time, or I have to stop for rests”, SGRQ13 sub-item 1 “I cannot play sports or games”, SGRQ14 “How does your chest trouble affect you?”.

| **Acquisition** | **Orientation** | **FOV** | **Slice thickness (mm)** | **Interval (mm)** | **Convolution kernel*** |
| --- | --- | --- | --- | --- | --- |
| **Inspiratory** | axial | lung | 1.25-1.50 | 0.70-0.75 | B70f/LUNG/L |
| **Inspiratory** | axial | lung | 0.625-1.00 | 0.50 | B30f/SOFT/B |
| **Inspiratory** | axial | including soft tissue of torso | 0.625-1.00 | 0.50 | B30f/SOFT/B |
| **Expiratory** | axial | lung | 0.625-1.00 | 0.50 | B30f/SOFT/B |

| **Scanner models** | Siemens Definition Siemens Definition AS 40/ 64/ Flash 128 GE Lightspeed VCT 64/ GE Optima 64/ Philips Brilliance 64/ iCT 256 |
| --- | --- |
|  |  |
| Scan Type | Spiral |
| Rotation Time (s) | 0.33 – 0.50 s |
| Collimation | 40 / 64 / 128 x 0.6-0.625 mm |
| Pitch | 0.6-1.0 |
| kVp | 120 kVp |
| mA | 30 - 35 eff. mAs |
| Dose modulation | Off |
| Matrix | 512 x 512 |
| Calibration phantom | Air / water phantom / CatPhan |
| Max. eff dose/scan | < 1.75 mSv |
| Max. eff. overall dose | < 3.50 mSv |

**Table S2. Technical details of the CT assessment.** The acquisition protocol is given in the upper part and details on the scanner models in the lower part of the table. *Vendor-specific generic names for Siemens/GE/Philips

| SGRQ item number | Item |
| --- | --- |
| 8 | How would you describe your chest condition? |
|  | a) Causes me a lot of problems or is the most important problem I have |
|  | b) Causes me a few problems |
|  | c) Causes no problem |
| 12, sub-item 3 | I walk slower than other people, or I stop for rests |

**Table S3. Items from the “St. George’s Respiratory Questionnaire” (SGRQ) that turned out to be informative in the single decision trees**

| CAT item number |  | Score |  | Short description of items |
| --- | --- | --- | --- | --- |
| 1 | I never cough | 0 1 2 3 4 5 | I cough all the time | Cough |
| 2 | I have no phlegm (mucus) on my chest at all | 0 1 2 3 4 5 | My chest is full of phlegm (mucus) | Phlegm |
| 3 | My chest does not feel tight at all | 0 1 2 3 4 5 | My chest feels very tight | Chest tightness |
| 4 | When I walk up a hill or a flight of stairs I am not out of breath | 0 1 2 3 4 5 | When I walk up a hill or a flight of stairs I am completely out of breath | Breathlessness |
| 5 | I am not limited to doing any activities at home | 0 1 2 3 4 5 | I am completely limited to doing any activities at home | Activities |
| 6 | I am confident leaving my home despite my lung condition | 0 1 2 3 4 5 | I am not confident leaving my home at all because of my lung condition | Confidence |
| 7 | I sleep soundly | 0 1 2 3 4 5 | I do not sleep soundly because of my lung condition | Sleep |
| 8 | I have lots of energy | 0 1 2 3 4 5 | I have no energy at all | Energy |

**Table S4. Items from the COPD Assessment test (CAT)**

| mMRC scale |  |
| --- | --- |
| 0 | Not troubled by breathlessness except on strenuous exercise |
| 1 | Short of breath when hurrying or walking up a slight hill |
| 2 | Walks slower than contemporaries on the level because of breathlessness or has to stop for breath when walking at own pace |
| 3 | Stops for breath after walking 100m or after a few minutes on the level |
| 4 | Too breathless to leave the house or breathless when dressing or undressing |

**Table S5. Modified Medical Research Council (mMRC) scale**. This self-rating questionnaire is used to measure the degree of disability that breathlessness poses on day-to-day activities on a scale from 0 to 4.

**Literature**

1 Biederer JWJ, B. H., Fink C, Tuengerthal S, Rehbock B. Protocol Recommendations for Computed Tomography of the Lung: Consensus of the Chest Imaging Workshop of the German Radiologic Society. *RöFo* **5**, 471-479 (2008).

2 Group, C. O. C. W. *et al.* A combined pulmonary-radiology workshop for visual evaluation of COPD: study design, chest CT findings and concordance with quantitative evaluation. *COPD* **9**, 151-159, doi:10.3109/15412555.2012.654923 (2012).
